# Non-melanoma skin cancer and risk of Alzheimer’s disease and all-cause dementia

**DOI:** 10.1371/journal.pone.0171527

**Published:** 2017-02-22

**Authors:** Sigrun A. J. Schmidt, Anne G. Ording, Erzsébet Horváth-Puhó, Henrik T. Sørensen, Victor W. Henderson

**Affiliations:** 1 Department of Clinical Epidemiology, Aarhus University Hospital, Aarhus, Denmark; 2 Department of Health Research & Policy (Epidemiology), Stanford University, Stanford, California, United States of America; 3 Department of Neurology & Neurological Sciences, Stanford University, Stanford, California, United States of America; Universiteit Antwerpen, BELGIUM

## Abstract

Cancer patients may be at decreased risk of Alzheimer’s disease. This hypothesis is best developed for non-melanoma skin cancer (NMSC), but supportive epidemiological data are sparse. We therefore conducted a nationwide cohort study of the association between NMSC and Alzheimer’s disease (main outcome) and all-cause dementia. Using Danish medical databases, we identified adults diagnosed with NMSC between 1 January 1980 and 30 November 2013 (n = 216,221) and a comparison cohort of five individuals matched to each NMSC patient by sex and birth year (n = 1,081,097). We followed individuals from the time of diagnosis, or corresponding date for matched comparators, until a dementia diagnosis, death, emigration, or 30 November 2013, whichever came first. We used stratified Cox regression adjusted for comorbidities to compute hazard ratios (HRs) associating NMSC with dementia. We computed cumulative risks of dementia, treating death as a competing risk. NMSC was associated with a HR of 0.95 (95% confidence interval [CI]: 0.92–0.98) for Alzheimer’s disease and 0.92 (95% CI: 0.90–0.94) for all-cause dementia. HRs were similar for basal cell and squamous cell carcinoma, the two most common forms of NMSC. Estimates of risk reduction were more pronounced in the beginning of follow-up, reaching null after 5–10 years. At the end of follow-up (34 years), cumulative risk of Alzheimer’s disease was 4.6% (95% CI: 4.4%–4.8%) among patients with NMSC *vs*. 4.7% (95% CI: 4.6%–4.9%) in the comparison cohort. In conclusion, NMSC was associated with 2%–10% reductions in relative risks of Alzheimer’s disease and all-cause dementia. However, these small inverse associations may have been caused by ascertainment bias due to decreased awareness of NMSC tumors in persons with undiagnosed early cognitive impairment or by confounding from a more neuroprotective lifestyle among persons with NMSC.

## Introduction

Persons with cancer may be at decreased risk of Alzheimer’s disease and other dementias [1,2]. Much of this inverse association may be explained by non-melanoma skin cancer (NMSC) [3–5]—the most frequent type of cancer in Western populations [6].

A number of plausible biological factors link cancer to a decline in incident dementia. For example, decreased activity of tumor suppressor proteins and overexpression of Pin1 (peptidyl-prolyl cis-trans isomerase, NIMA-interacting 1) are associated both with cancer and with a lower risk of Alzheimer’s disease [2,7]. Furthermore, the major risk factor for NMSC, ultraviolet radiation exposure [8], boosts vitamin D levels, which may protect against dementia [8].

Epidemiological studies on the association between NMSC and dementia are sparse. In a cohort study conducted among participants in the Einstein Aging Study in New York, the hazard ratio (HR) of all-cause dementia among individuals with self-reported NMSC was reduced by over one-third compared with participants without this cancer, with even larger reductions for Alzheimer’s disease [3]. Although this study was limited to volunteers aged 70 years or older followed for a median of three years, others have reported similar results [5]. Findings may reflect shared biological mechanisms in both NMSC and Alzheimer’s disease that operate in opposite directions, but bias or unrecognized confounding should also be considered for these associations.

To provide more accurate estimates of dementia risk within a general population cohort with long-term follow-up, we conducted a large matched cohort study of the association between NMSC and risk of Alzheimer’s disease (primary outcome) and all-cause dementia.

## Materials and methods

### Settings and data sources

We conducted this study in Denmark, which has a population of 5.6 million persons. The Danish government provides tax-supported universal health care [9]. Administrative and medical data for all residents are recorded in nationwide databases. Individual-level linkage of the databases is possible using a unique personal identifier assigned to all residents by the Danish Civil Registration System since 1968 [9]. This registry also records daily changes in vital status and migration for the entire population.

The Danish National Patient Registry (DNPR) and the Danish Psychiatric Central Research Register (PCRR) are population-based hospital registries, which were linked in 1995 [10]. Together they provide nationwide coverage of all inpatient psychiatric contacts since 1970, all inpatient non-psychiatric contacts since 1978, and all hospital outpatient specialty clinic visits and emergency department visits since 1995 [10]. For each inpatient and outpatient hospital contact, patient identifiers, dates of admission and discharge or start and end of outpatient follow-up, diagnoses, and treatments are recorded [10]. At the time of discharge or hospital contact, the treating physician is responsible for coding a primary diagnosis (the main reason for the hospital contact) and, when relevant, one or more secondary diagnoses, using the *International Classification of Diseases*, *Eighth Revision* (ICD-8) until the end of 1993 and the *Tenth Revision* (ICD-10) thereafter. General practitioners and private practice specialists do not submit data to the hospital registries.

The Danish Cancer Registry (DCR) records data on all incident cancers, including diagnoses made at departments of pathology and forensic medicine [11]. Reporting to the DCR began on a manual basis in 1943, supplemented with data from the Registry of Causes of Deaths (1943–) and the DNPR (1987–) [11]. Since a modernization process was initiated in 2004, the DCR has combined data reported electronically to the DNPR, the National Pathology Register, and the Registry of Causes of Death, and directly from general practitioners, private practice specialists, and private hospitals. For the current study period, data were coded according to ICD-10 and the third version of ICD for Oncology (ICD-O-3).

### Study population

From a source population of 6.9 million persons, we identified all adults aged 18 years or older with a first-time NMSC recorded in the DCR between 1 January 1980 and 30 November 2013. Our definition of NMSC included basal cell carcinoma, squamous cell carcinoma, and other rare subtypes. Persons diagnosed with both basal cell and squamous cell carcinomas on the same date were categorized in the latter group. We did not include patients with a previous diagnosis of dementia or of mild cognitive impairment or an amnestic syndrome (which may represent early symptoms of an underlying dementing disorder) in the DNPR or PCRR.

We used the Civil Registration System to randomly select a comparison cohort of up to five individuals matched (with replacement) to each NMSC patient by sex and birth year. Persons in the matched cohort were alive and without a previous diagnosis of NMSC, dementia, mild cognitive impairment, or amnestic syndrome on the index date (defined as the diagnosis date in the DCR for the matched NMSC patient).

Registry codes used in this study are provided in S1 Table.

### Dementia

We used the DNPR and the PCRR to identify all inpatient and hospital outpatient clinic diagnoses of dementia among study participants following the index date. The hospital admission date or start date of outpatient clinic follow-up was considered the date of dementia diagnosis. We subcategorized codes for all-cause dementia Alzheimer’s disease, vascular dementia, or other dementia (S1 Table). Our hypothesis was that NMSC would be associated with a reduced risk of Alzheimer’s disease.

### Comorbidity

We identified participants’ history of hospital inpatient and outpatient diagnoses in the DNPR before or on the index date. We included diagnoses of cardiovascular diseases, including hypertension, ischemic heart disease (angina pectoris, myocardial infarction, or percutaneous coronary intervention), congestive heart failure, and peripheral artery disease. As proxies of cardiovascular risk factors (*e*.*g*., obesity and smoking), we also identified diagnoses of hospital-based obesity, diabetes, and chronic obstructive pulmonary disease. Similarly, we included hospital contacts related to excessive alcohol use, since alcohol consumption is associated with risk of cutaneous cancer [12] and perhaps dementia [13]. We also identified any history of cancer and multiple sclerosis, as they may be associated with both dementia [2,14] and NMSC [15,16]. Finally, we retrieved diagnoses of rare risk factors for NMSC, including solid organ transplantation, human immunodeficiency virus infection, xeroderma pigmentosum, nevoid basal cell carcinoma syndrome, and albinism. The hospital discharge date or end of outpatient follow-up was considered the diagnosis date for all comorbidities.

### Statistical analysis

We followed participants from the index date until a diagnosis of dementia, death, emigration, or 30 November 2013, whichever came first. Comparison cohort members who were diagnosed with NMSC during follow-up (4.3% of the matched cohort) were censored and included in the NMSC cohort on the date of diagnosis.

We computed rates of dementia and used Cox proportional hazard regression stratified by matching factors to compute unadjusted HRs and 95% confidence intervals (CIs) of all-cause dementia and dementia subtypes in the NMSC cohort compared with the matched cohort. We then also adjusted for the individual cardiovascular diseases, risk factors for cardiovascular diseases, alcohol-related diagnoses, cancer, and multiple sclerosis. Considering death as a competing risk, we plotted the cumulative risk of dementia for each cohort and expressed the risk at pre-specified time periods (one year, five years, ten years, and the overall follow-up period). We analyzed the association with dementia for NMSC overall (our primary predictor) and for basal cell and squamous cell carcinoma separately.

We performed several predefined subgroup analyses. First, we examined whether associations varied by age at NMSC diagnosis (18–49, 50–59, 60–74, 75–84, ≥85 years) or sex. Second, because data quality may have been affected by registry changes, such as inclusion of outpatient and emergency departments in the DNPR and modernization of the DCR, we stratified results by relevant calendar periods for index date (1980–1994, 1995–2003, 2004–2013). Finally, we examined whether associations depended on the presence or absence of dementia risk factors, including any cardiovascular diseases or risk factors, alcohol-related diagnoses, and other cancers. Because matching could not be retained when stratifying by comorbidities, we used conventional Cox regression with additional adjustment for age, sex, and calendar period of the index date.

We examined the robustness of our results in several sensitivity analyses. First, we repeated analyses after excluding persons with a previous diagnosis of cancer and the aforementioned rare risk factors of NMSC. Second, we redefined Alzheimer’s disease by including unspecified diagnoses of dementia, as about a third of such diagnoses are known to fulfill criteria for Alzheimer’s disease [17]. This sensitivity analysis would increase the completeness of ascertainment of Alzheimer’s disease, but at the cost of a decreased positive predictive value (PPV). Third, to increase the comparability of our study with the Einstein Aging Study [4], we adjusted for alcohol-related and cardiovascular comorbidity as time-varying covariates, rather than relying only on baseline information.

We tested the assumption of proportional hazards using plots of the log(-log(survival)) *vs*. log of survival time, which revealed disproportionality between cohorts, mainly during the initial follow-up period. In a post hoc analysis, we therefore stratified the results by follow-up time (0–1 year, >1–5 years, >5–10 years, >10–34 years).

All analyses were performed using SAS 9.2^®^ (SAS Institute Inc., Cary, NC, U.S.A.).

### Ethics

The study was approved by the Danish Data Protection Agency (2014-54-0922). Danish legislation does not require ethical review board approval or informed consent from subjects in registry-based studies.

## Results

We identified 216,221 patients with NMSC (84.3% basal cell carcinoma, 11.9% squamous cell carcinoma, and 3.8% other types) and 1,081,097 matched individuals. The median age was 68 years (interquartile range: 58–78 years) and 51% were women (Table 1). The majority of patients were diagnosed in the most recent calendar period. A history of other cancers was more frequent among NMSC patients than in the comparison cohort. Examination of patient characteristics according to NMSC subtype revealed that the baseline prevalence of other comorbidities was slightly higher among patients with squamous cell carcinoma and slightly lower among patients with basal cell carcinoma when compared to their respective comparison cohorts (S2 Table).

**Table 1:** Selected characteristics of persons diagnosed with non-melanoma skin cancer and members of a matched comparison cohort, Denmark, 1980–2013.

	Non-melanoma skin cancer (n = 216,221)	Comparison cohort (n = 1,081,097)
**Age groups, years**		
18–49	27,445 (12.7%)	137,326 (12.7%)
50–59	34,623 (16.0%)	173,317 (16.0%)
60–74	84,828 (39.2%)	424,042 (39.2%)
75–84	49,531 (22.9%)	247,422 (22.9%)
85–106	19,794 (9.2%)	98,990 (9.2%)
**Sex**		
Women	110,235 (51.0%)	551,173 (51.0%)
Men	105,986 (49.0%)	529,924 (49.0%)
**Calendar period of non-melanoma skin cancer diagnosis**		
1980–1994	54,261 (25.1%)	271,301 (25.1%)
1995–2003	49,626 (23.0%)	248,128 (23.0%)
2004–2013	112,334 (52.0%)	561,668 (52.0%)
**Comorbidities**		
Hospital-diagnosed obesity	3,799 (1.8%)	23,925 (2.2%)
Hypertension	23,736 (11.0%)	109,832 (10.2%)
Ischemic heart disease	17,817 (8.2%)	89,349 (8.3%)
Angina pectoris	13,057 (6.0%)	63,432 (5.9%)
Myocardial infarction	8,950 (4.1%)	47,598 (4.4%)
Percutaneous coronary intervention	3,416 (1.6%)	17,225 (1.6%)
Congestive heart failure	7,097 (3.3%)	36,137 (3.3%)
Peripheral artery disease	7,272 (3.4%)	35,988 (3.3%)
Diabetes	8,645 (4.0%)	49,235 (4.6%)
Chronic obstructive pulmonary disease	8,584 (4.0%)	45,768 (4.2%)
Alcohol-related disease	3,132 (1.4%)	20,207 (1.9%)
Other cancer	23,547 (10.9%)	84,399 (7.8%)
Multiple sclerosis	492 (0.2%)	2,364 (0.2%)
Solid organ transplantation	484 (0.2%)	275 (0.0%)
Human immunodeficiency virus infection	134 (0.1%)	341 (0.0%)
**Follow-up (years)**		
Total (range)	1,665,541 (0–34)	8,134,184 (0–34)
Median (interquartile range)	5.8 (2.6–10.9)	5.6 (2.5–10.6)

Data are numbers (%). Xeroderma pigmentosum, nevoid basal cell carcinoma syndrome, and albinism were omitted from the table because of low prevalence (less than three persons).

We followed NMSC and comparison cohorts for a median of 5.8 and 5.6 years, respectively (Table 1). Patients with squamous cell carcinoma had a shorter median follow-up than their comparators (4.3 *vs*. 4.7 years), whereas the opposite was observed for basal cell carcinoma patients and their comparison cohort (6.1 *vs*. 5.8 years).

In total, 4,179 persons in the NMSC cohort and 20,975 persons in the matched comparison cohort were diagnosed with incident Alzheimer’s disease. NMSC was associated with an adjusted HR of 0.95 (95% CI: 0.92–0.98) for Alzheimer’s disease and 0.92 (95% CI: 0.90–0.94) for all-cause dementia, without variation by subtype of skin cancer (Table 2). In analyses examining the association according to time periods since NMSC diagnosis, we found that estimates were most pronounced in the beginning of follow-up, reaching null after 5–10 years (Table 3). HRs for the secondary outcomes of vascular dementia and other non-Alzheimer’s dementias were 0.87 (95% CI: 0.82–0.92) and 0.92 (95% CI: 0.89–0.95), respectively (S3 Table).

**Table 2:** Rates and hazard ratios of dementia in patients with non-melanoma skin cancer compared with members of a matched comparison cohort, Denmark, 1980–2013.

	NMSC cohort		Comparison cohort		Unadjusted HR (95% CI)†	Adjusted HR (95% CI)‡
	No. of events	Rate (95% CI)*	No. of events	Rate (95% CI)*		
**Any NMSC**						
Alzheimer’s disease	4,179	2.51 (2.43–2.59)	20,975	2.58 (2.54–2.61)	0.95 (0.91–0.98)	0.95 (0.92–0.98)
All-cause dementia	11,681	7.01 (6.89–7.14)	59,667	7.34 (7.28–7.39)	0.92 (0.90–0.94)	0.92 (0.90–0.94)
**Basal cell carcinoma**						
Alzheimer’s disease	3,506	2.39 (2.31–2.47)	17,136	2.43 (2.39–2.47)	0.94 (0.91–0.98)	0.95 (0.91–0.98)
All-cause dementia	9,745	6.65 (6.52–6.78)	48,631	6.90 (6.84–6.96)	0.91 (0.89–0.94)	0.92 (0.90–0.94)
**Squamous cell carcinoma**						
Alzheimer’s disease	564	3.67 (3.37–3.98)	3,160	3.85 (3.72–3.99)	0.95 (0.86–1.05)	0.95 (0.86–1.05)
All-cause dementia	1,634	10.64 (10.12–11.16)	9,228	11.26 (11.03–11.49)	0.94 (0.88–0.99)	0.94 (0.88–0.99)

Abbreviations: CI = confidence interval; HR = hazard ratio; NMSC = non-melanoma skin cancer

* Rate per 1,000 person-years.

^†^Computed using stratified Cox proportional hazard regression adjusted by study design for age, sex, and calendar period of the skin cancer diagnosis/index date.

^‡^Adjusted additionally for alcohol-related diagnoses, hospital-diagnosed obesity, hypertension, ischemic heart disease (angina pectoris, myocardial infarction, and percutaneous coronary intervention), congestive heart failure, peripheral artery disease, chronic pulmonary disease, diabetes, cancer, and multiple sclerosis

**Table 3:** Adjusted hazard ratios (95% confidence intervals)* of dementia in patients with non-melanoma skin cancer compared with members of a matched comparison cohort, by length of follow-up, Denmark, 1980–2013.

	Follow-up time			
	0–1 year	>1–5 years	>5–10 years	>10–34 years
**Any NMSC**				
Alzheimer’s disease	0.88 (0.79–0.97)	0.90 (0.85–0.96)	0.98 (0.91–1.05)	1.03 (0.96–1.11)
All-cause dementia	0.87 (0.82–0.93)	0.89 (0.85–0.92)	0.94 (0.90–0.98)	0.98 (0.94–1.02)
**Basal cell carcinoma**				
Alzheimer’s disease	0.83 (0.74–0.94)	0.90 (0.84–0.96)	0.99 (0.91–1.07)	1.02 (0.95–1.10)
All-cause dementia	0.84 (0.78–0.91)	0.87 (0.83–0.91)	0.95 (0.91–1.00)	0.98 (0.93–1.02)
**Squamous cell carcinoma**				
Alzheimer’s disease	1.04 (0.83–1.29)	0.88 (0.75–1.02)	0.93 (0.76–1.14)	1.14 (0.88–1.47)
All-cause dementia	0.93 (0.81–1.08)	0.94 (0.86–1.03)	0.89 (0.79–1.00)	1.00 (0.86–1.16)

Abbreviations: NMSC = non-melanoma skin cancer

* Adjusted for alcohol-related diagnoses, hospital-diagnosed obesity, hypertension, ischemic heart disease (angina pectoris, myocardial infarction, and percutaneous coronary intervention), congestive heart failure, peripheral artery disease, chronic pulmonary disease, diabetes, cancer, and multiple sclerosis. Computed using stratified Cox proportional hazard regression adjusted by study design for age, sex, and calendar period of the skin cancer diagnosis/index date.

**Fig 1:**
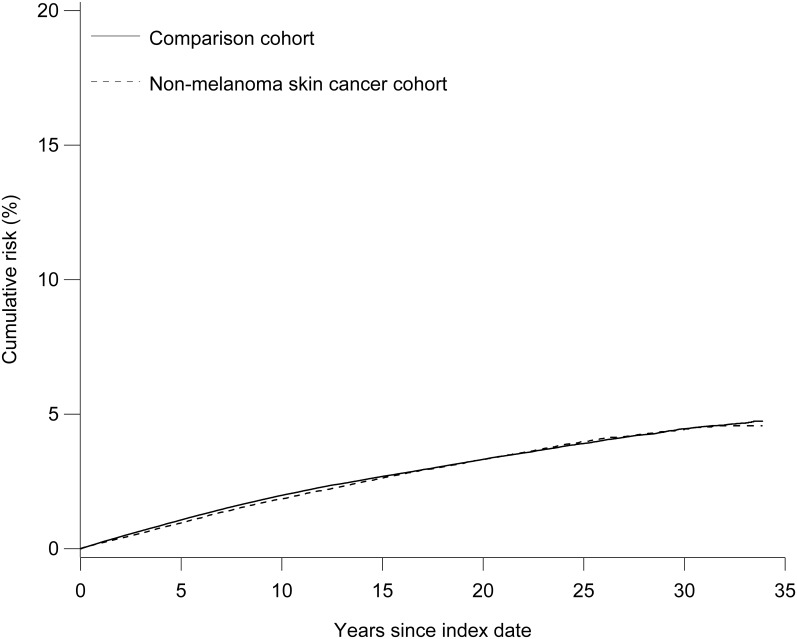
Cumulative risk of Alzheimer’s disease among patients with non-melanoma skin cancer and their comparison cohort.

**Fig 2:**
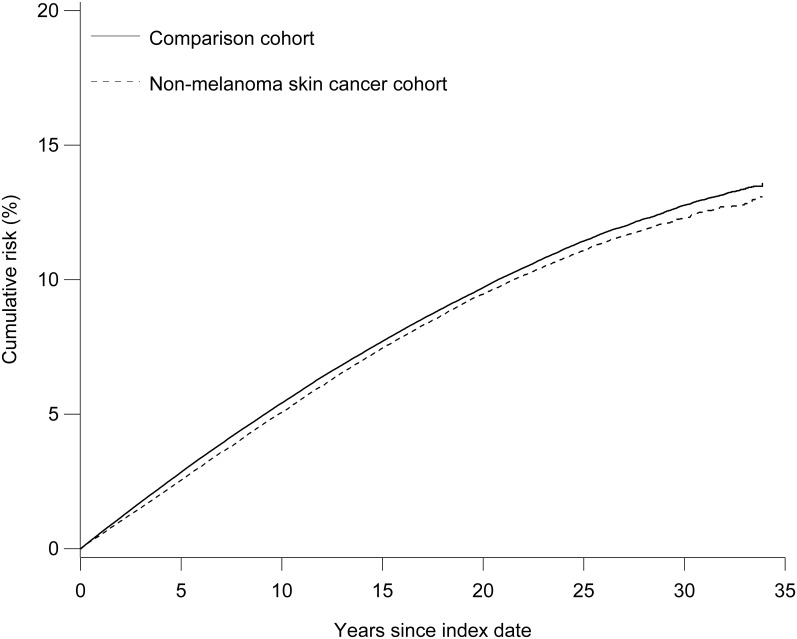
Cumulative risk of all-cause dementia among patients with non-melanoma skin cancer and their comparison cohort.

The cumulative risks of Alzheimer’s disease and all-cause dementia in the study cohorts are shown in Figs 1 and 2. There was no overall difference in the risk of Alzheimer’s disease in the NMSC (4.6%, 95% CI: 4.4%–4.8%) and comparison cohorts (4.7%, 95% CI: 4.6%–4.9%) at the end of follow-up (34 years). This finding was consistent with the cumulative risks observed in the basal cell carcinoma cohort (4.7%, 95% CI: 4.5%–5.0%) and its comparison cohort (4.8%, 95% CI: 4.7%–5.0%). However, persons with squamous cell carcinoma (3.8%, 95% CI: 3.4%–4.2%) had a lower risk of Alzheimer’s disease than persons in their comparison cohort (4.5%, 95% CI: 4.3%–4.7%).

We observed no major differences in associations between NMSC and Alzheimer’s disease (Table 4) or all-cause dementia (S4 Table) in subgroups defined by age, sex, calendar period of NMSC diagnosis, and comorbidities. The HRs also remained virtually unchanged after excluding persons with NMSC risk factors (S5 Table) and after using time-dependent rather than baseline adjustment for covariates (S6 Table). Classification of unspecified dementia as Alzheimer’s disease resulted in more than twice as many events in the cohorts without a substantial effect on the HRs (S7 Table).

**Table 4:** Rates and hazard ratios of Alzheimer’s disease in patients with non-melanoma skin cancer compared with members of a matched comparison cohort, by selected characteristics, Denmark, 1980–2013.

	Rate (95% CI) per 1,000 person-years		Unadjusted HR (95% CI)*		Adjusted HR (95% CI)†
	NMSC cohort	Comparison cohort			
**Age (years)**					
18–49	0.09 (0.06–0.13)	0.12 (0.10–0.14)	0.76 (0.50–1.15)		0.76 (0.50–1.15)
50–59	0.67 (0.58–0.75)	0.64 (0.60–0.67)	1.03 (0.88–1.20)		1.02 (0.87–1.19)
60–74	2.53 (2.41–2.65)	2.48 (2.42–2.53)	0.98 (0.93–1.04)		0.99 (0.93–1.05)
75–84	6.41 (6.11–6.71)	6.86 (6.72–7.01)	0.94 (0.89–0.99)		0.94 (0.89–1.00)
85+	7.10 (6.47–7.74)	8.27 (7.96–8.59)	0.86 (0.78–0.96)		0.87 (0.78–0.96)
**Sex**					
Women	2.70 (2.59–2.80)	2.84 (2.79–2.89)	0.92 (0.88–0.96)		0.92 (0.88–0.97)
Men	2.30 (2.19–2.41)	2.29 (2.24–2.34)	0.99 (0.93–1.04)		0.99 (0.93–1.04)
**Calendar period of NMSC diagnosis**					
1980–1994	2.57 (2.45–2.69)	2.56 (2.51–2.61)	0.99 (0.93–1.04)		0.99 (0.93–1.04)
1995–2003	2.44 (2.30–2.57)	2.41 (2.35–2.47)	0.96 (0.90–1.03)		0.97 (0.90–1.03)
2004–2013	2.49 (2.35–2.64)	2.79 (2.72–2.86)	0.88 (0.82–0.94)		0.89 (0.83–0.95)
**Any history of cardiovascular diseases or risk factors**					
Yes	3.52 (3.28–3.75)	3.70 (3.59–3.81)		0.90 (0.84–0.97)	0.91 (0.84–0.98)
No	2.34 (2.26–2.42)	2.39 (2.35–2.43)		0.96 (0.92–0.99)	0.96 (0.92–0.99)
**Any history of alcohol-related disease**					
Yes	1.41 (0.83–1.99)	1.79 (1.53–2.05)		0.71 (0.46–1.09)	0.71 (0.46–1.10)
No	2.52 (2.44–2.60)	2.59 (2.55–2.62)		0.95 (0.92–0.98)	0.95 (0.92–0.98)
**Any history of other cancer**					
Yes	3.09 (2.78–3.40)	3.66 (3.47–3.85)		0.91 (0.82–1.02)	0.91 (0.82–1.02)
No	2.46 (2.38–2.54)	2.52 (2.49–2.56)		0.95 (0.92–0.98)	0.95 (0.92–0.98)

Abbreviations: CI = confidence interval; HR = hazard ratio; NMSC = non-melanoma skin cancer

* Computed using stratified Cox proportional hazard regression adjusted by study design for age, sex, and calendar period of skin cancer diagnosis/index date. In analyses stratified by comorbidities, conventional Cox regression was used with additional adjustment for matching factors.

^†^ Adjusted additionally for alcohol-related diagnoses, hospital-diagnosed obesity, hypertension, ischemic heart disease (angina pectoris, myocardial infarction, and percutaneous coronary intervention), congestive heart failure, peripheral artery disease, chronic pulmonary disease, diabetes, other cancer, and multiple sclerosis.

## Discussion

Using registry data collected over a period of more than 40 years, we found that NMSC was associated with small reductions in relative risks of Alzheimer’s disease (5%) and all-cause dementia (8%), compared to individuals without NMSC. The findings were explained by decreased HRs within the first five years after diagnosis or corresponding data among matched comparators. The absolute risk difference of Alzheimer’s disease was below 1% after ten years of follow-up.

The reductions observed in our study are considerably smaller than previously reported. In the Einstein Aging Study, which included 1,102 older adults, NMSC was associated with substantial decreases in risk of all-cause dementia (HR 0.64, 95% CI: 0.32–1.23), driven primarily by the results for Alzheimer’s disease (HR 0.20, 95% CI: 0.05–0.81) [3]. However, the number of observed outcomes was modest (126 persons developed all-cause dementia and 76 developed Alzheimer’s disease), and analyses were based on a mix of prevalent (77%) cases and incident (23%) cases of self-reported of NMSC. Among volunteers in the Alzheimer’s Disease Neuroimaging Initiative, a self-reported or informant-reported history of prior NMSC was a third less common at baseline among Alzheimer’s cases than participants without dementia [5]. Participants without baseline dementia were more likely to develop Alzheimer’s disease when there was no reported history of NMSC [5].

One inference from the previously reported inverse associations between NMSC and dementia or Alzheimer’s disease is that these conditions share genetic or biological mechanisms that work in opposite directions. Findings may be similar for other forms of cancer as well [1,2]. Indeed, cancer and the neurodegenerative dementias have been conceptualized as involving polar processes leading towards unbridled proliferation at one extreme and apoptosis at the other [2]. One would not necessarily predict common mechanisms in vascular dementia, however [18], where we also observed reduced risk after NMSC. Possible bias and confounding should thus also be considered [2]. It is possible that physicians are less likely to pursue a dementia diagnosis in patients battling or recovering from cancer, or conversely, that NMSC remains undetected in a person with early cognitive impairment (*i*.*e*., ascertainment bias) [2]. Our findings of more pronounced estimates in the beginning of follow-up provide support for the latter. Furthermore, cancer patients may not survive long enough to develop dementia [2]. Although survivor bias is less problematic for NMSC than for more aggressive forms of cancer, squamous cell carcinoma is linked to increased mortality, attributed to respiratory, cardiovascular, and infectious diseases [19]. Indeed, compared with the general population comparison cohorts, median survival in our study was shorter in the squamous cell cancer cohort, but not the basal cell carcinoma cohort. The more apparent reductions in cumulative risk of dementia (compared with HRs) associated with squamous cell carcinoma but not basal cell carcinoma may reflect this slight disadvantage in survival, as such effects are evident in competing risk analyses but not Cox proportional hazard regression models.

In our study, we used population-based registries to identify an inception cohort of NMSC patients diagnosed in a universal healthcare system. This approach limits potential problems with selection bias, sample size, and generalizability. In particular, the exclusion of prevalent cases reduced the likelihood of survivor bias, which can imply spurious protective effects [20]. Furthermore, our study had a longer follow-up period than previous studies, with more accurate dates of diagnosis and censoring events [9,10]. We were also able to stratify analyses according to subtypes of NMSC. Such analyses are important for elucidation of potential underlying biological mechanisms, which differ for basal cell and squamous cell carcinoma [6,21]. We found no major differences in HRs, which suggest that, if valid, mechanisms driving the observed associations are common to both types.

Our study also has several limitations. Based on the number of NMSCs reported by other Danish data sources, it has been estimated that roughly 20%–55% of these cancers are not included in the DCR [22–24]. However, this estimated level of non-inclusion may be too high, because many patients have recurrent NMSC cancers and the DCR records skin cancers with the same morphology only once for each patient [25]. To introduce selection bias in our study, the association between skin cancer and dementia would additionally have to differ among unreported and reported cases, which we consider implausible. Furthermore, our results did not vary by calendar period despite the expected improvement in data quality following modernization of the DCR in 2004.

The validity of diagnoses of all-cause dementia and Alzheimer’s disease in Danish registries is high, except for uncommon and early-onset variants [17,26]. For example, one study reported PPVs of 81% for Alzheimer’s disease and 86% for all-cause dementia when information from medical records was compared with patient interviews (available for 25% of the patient sample) using ICD-10 and/or Diagnostic and Statistical Manual of Mental Disorders (DSM)-IV diagnosis criteria [17]. As history of NMSC would be unlikely to lower the chance of having Alzheimer’s disease coded and because our results were similar for Alzheimer’s disease and other dementia diagnoses, which are known to have lower PPVs [17], we do not believe that misclassification had substantial impact on our results. Incomplete reporting of potentially milder cases from primary healthcare providers and private practice specialists (estimated one-third of dementia diagnoses [27]) may affect the generalizability of our results, but only if the association between NMSC and dementia or Alzheimer’s disease depends on dementia severity.

Although adjustment for various comorbidities did not affect our results, residual confounding from lifestyle factors linked to cognitive decline may explain the slight risk reductions observed. For example, physical activity is associated with lower risks of dementia and Alzheimer’s disease [13] but is also associated with ultraviolet radiation exposure—and hence NMSC—if activities are performed outdoors [6]. Furthermore, basal cell carcinoma, and possibly squamous cell carcinoma, is diagnosed more frequently in people with higher socioeconomic status [28,29], who are typically better educated and have healthier lifestyles [30]. We also lacked data on genetic susceptibility to dementia. However, adjustment for the number of apolipoprotein E ε4 alleles did not materially affect previously reported associations [3].

## Conclusions

This large nationwide study suggests modest inverse associations between NMSC and Alzheimer’s disease and all-cause dementia. If these conditions share common biological mechanisms, further research of NMSC patients could provide clues into novel neuroprotective mechanisms of Alzheimer’s disease. However, the observed small effect sizes, evidence for ascertainment bias, and the possibility of residual confounding from lifestyle factors associated with both Alzheimer’s risk and NMSC suggest the alternative interpretation that NMSC is unrelated to either Alzheimer’s disease or all-cause dementia.

## Supporting information

S1 Strobe Checklist of items that should be included in reports of cohort studies. (DOC)

S1 Table Registry codes used in the study. (DOCX)

S2 Table Selected characteristics of persons diagnosed with non-melanoma skin cancer and members of a matched comparison cohort, Denmark, 1980–2013. (DOCX)

S3 Table Rates and hazard ratios of vascular dementia and other non-Alzheimer dementia in patients with non-melanoma skin cancer compared with a matched comparison cohort, Denmark 1980–2013. (DOCX)

S4 Table Rates and hazard ratios of all-cause dementia associated with a previous diagnosis of non-melanoma skin cancer, by study characteristics, Denmark 1980–2013. (DOCX)

S5 Table Adjusted hazard ratios (95% confidence intervals)* of dementia associated with a previous diagnosis of non-melanoma skin cancer, Denmark 1980–2013. Sensitivity analysis excluding persons with previous cancer diagnosis, solid organ transplantation, HIV infection, xeroderma pigmentosum, nevoid basal cell carcinoma syndrome, and/or albinism.
(DOCX)

S6 Table Adjusted hazard ratios (95% confidence interval)* of dementia associated with a previous diagnosis of non-melanoma skin cancer, Denmark 1980–2013. Sensitivity analysis adjusting for alcohol-related disease and cardiovascular diseases and risk factors as time-varying covariates.
(DOCX)

S7 Table Rates and hazard ratios of Alzheimer disease in patients with non-melanoma skin cancer compared with members of a matched comparison cohort, Denmark, 1980–2013. Sensitivity analysis including unspecified dementia diagnoses in the classification of Alzheimer disease*.
(DOCX)

## Abbreviations

CI: confidence interval
DCR: Danish Cancer Registry
DNPR: Danish National Patient Registry
HR: hazard ratio
ICD: International Classification of Diseases
NMSC: non-melanoma skin cancer
PCRR: Danish Psychiatric Central Research Register
Pin1: peptidyl-prolyl cis-trans isomerase, NIMA-interacting 1
PPV: positive predictive value

## References

[1] Ma L-L, Yu J-T, Wang H-F, Meng X-F, Tan C-C, Wang C, et al Association between cancer and Alzheimer's disease: systematic review and meta-analysis. J Alzheimers Dis. 2014;42: 565–573. 10.3233/JAD-140168 24906231

[2] Ganguli M. Cancer and Dementia: It's Complicated. Alzheimer Dis Assoc Disord. 2015. 10.1097/WAD.0000000000000086 PMC4437917 25710132

[3] White RS, Lipton RB, Hall CB, Steinerman JR. Nonmelanoma skin cancer is associated with reduced Alzheimer disease risk. Neurology. 2013;80: 1966–1972. 10.1212/WNL.0b013e3182941990 23677746 PMC3716346

[4] Behrens MI, Roe C, Morris JC. Inverse association between cancer and dementia of the Alzheimer’s type In: Bernhardi von R, Inestrosa NC, editors. Neurodegenerative Diseases: From Molecular Concepts to Therapeutic Targets. New York: Nova Science Publishers; 2008 pp. 111–120.

[5] Nudelman KNH, Risacher SL, West JD, McDonald BC, Gao S, Saykin AJ, et al Association of cancer history with Alzheimer's disease onset and structural brain changes. Front Physiol. 2014;5: 423 10.3389/fphys.2014.00423 25400589 PMC4215790

[6] Madan V, Lear JT, Szeimies R-M. Non-melanoma skin cancer. Lancet. Elsevier Ltd; 2010;375: 673–685. 10.1016/S0140-6736(09)61196-X 20171403

[7] Behrens MI, Lendon C, Roe CM. A common biological mechanism in cancer and Alzheimer's disease? Curr Alzheimer Res. 2009;6: 196–204. 19519301 10.2174/156720509788486608 PMC2810550

[8] Annweiler C, Dursun E, Féron F, Gezen-Ak D, Kalueff AV, Littlejohns T, et al “Vitamin D and cognition in older adults”: updated international recommendations. J Intern Med. 2015;277: 45–57. 10.1111/joim.12279 24995480

[9] Schmidt M, Pedersen L, Sørensen HT. The Danish Civil Registration System as a tool in epidemiology. Eur J Epidemiol. 2014;29: 541–549. 10.1007/s10654-014-9930-3 24965263

[10] Schmidt M, Schmidt SAJ, Sandegaard JL, Ehrenstein V, Pedersen L, Sørensen HT. The Danish National Patient Registry: A review of content, data quality, and research potential. Clin Epidemiol. 2015;7: 449–490. 10.2147/CLEP.S91125 26604824 PMC4655913

[11] Gjerstorff ML. The Danish Cancer Registry. Scand J Public Health. 2011;39: 42–45. 10.1177/1403494810393562 21775350

[12] Kubo JT, Henderson MT, Desai M, Wactawski-Wende J, Stefanick ML, Tang JY. Alcohol consumption and risk of melanoma and non-melanoma skin cancer in the Women's Health Initiative. Cancer Causes Control. 2013;25: 1–10. 10.1007/s10552-013-0280-3 24173533 PMC5515083

[13] Prince M, Albanese E, Guerchet M, Prina M. World Alzheimer report 2014. Dementia and risk reduction: an analysis of protective and modifiable factors [Internet]. Alzheimers Disease International; 2014. http://www.alz.co.uk/research/WorldAlzheimerReport2014.pdf

[14] Robinson L, Tang E, Taylor J-P. Dementia: timely diagnosis and early intervention. BMJ. 2015;350: h3029 10.1136/bmj.h3029 26079686 PMC4468575

[15] Jensen AO, Olesen AB, Dethlefsen C, Sorensen HT, Karagas MR. Chronic diseases requiring hospitalization and risk of non-melanoma skin cancers—a population based study from Denmark. J Invest Dermatol. 2008;128: 926–931. 10.1038/sj.jid.5701094 17914446

[16] Bahmanyar S, Montgomery SM, Hillert J, Ekbom A, Olsson T. Cancer risk among patients with multiple sclerosis and their parents. Neurology. 2009;72: 1170–1177. 10.1212/01.wnl.0000345366.10455.62 19332695

[17] Phung TKT, Andersen BB, Høgh P, Kessing LV, Mortensen PB, Waldemar G. Validity of dementia diagnoses in the Danish hospital registers. Dement Geriatr Cogn Disord. 2006;24: 220–228. 10.1159/000107084 17690555

[18] Roe CM, Fitzpatrick AL, Xiong C, Sieh W, Kuller L, Miller JP, et al Cancer linked to Alzheimer disease but not vascular dementia. Neurology. AAN Enterprises; 2010;74: 106–112. 10.1212/WNL.0b013e3181c91873 PMC2809029 20032288

[19] Jensen AØ, Bautz A, Olesen AB, Karagas MR, Sørensen HT, Friis S. Mortality in Danish patients with nonmelanoma skin cancer, 1978–2001. Br J Dermatol. 2008;159: 419–425. 10.1111/j.1365-2133.2008.08698.x 18616784 PMC3255323

[20] Saracci R. Survival-related biases survive well. Int J Epidemiol. 2007;36: 244–246. 10.1093/ije/dyl263 17169941

[21] English DR, Armstrong BK, Kricker A, Fleming C. Sunlight and cancer. Cancer Causes Control. 1997;8: 271–283. 9498892 10.1023/a:1018440801577

[22] Jensen AØ, Olesen AB, Dethlefsen C, Sørensen HT. Do incident and new subsequent cases of non-melanoma skin cancer registered in a Danish prospective cohort study have different 10-year mortality? Cancer Detect Prev. 2007;31: 352–358. 10.1016/j.cdp.2007.04.011 18031945

[23] Frentz G. General skin cancer. Quantity, treatment and quality (Danish). Ugeskr Laeger. 1996;158: 7202 9012031

[24] Frentz G, Olsen JH. Malignant tumours and psoriasis: a follow-up study. Br J Dermatol. 1999;140: 237–242. 10233215 10.1046/j.1365-2133.1999.02655.x

[25] Brandt B, Møller-Hansen K, Fajber M. Cancer Incidence in Denmark 2000 The Danish National Board of Health. 2004;: 1–85. http://www.sst.dk

[26] Salem LC, Andersen BB, Nielsen TR, Stokholm J, Jørgensen MB, Rasmussen MH, et al Overdiagnosis of dementia in young patients—a nationwide register-based study. Dement Geriatr Cogn Disord. 2011;34: 292–299. 10.1159/000345485 23208125

[27] Phung TKT, Waltoft BL, Kessing LV, Mortensen PB, Waldemar G. Time trend in diagnosing dementia in secondary care. Dement Geriatr Cogn Disord. 2009;29: 146–153. 10.1159/000269933 20150733

[28] Steding-Jessen M, Birch-Johansen F, Jensen A, Schüz J, Kjaer SK, Dalton SO. Socioeconomic status and non-melanoma skin cancer: a nationwide cohort study of incidence and survival in Denmark. Cancer Epidemiol. 2010;34: 689–695. 10.1016/j.canep.2010.06.011 20638927

[29] Asgari MM, Efird JT, Warton EM, Friedman GD. Potential risk factors for cutaneous squamous cell carcinoma include oral contraceptives: results of a nested case-control study. Int J Environ Res Public Health. 2010;7: 427–442. 10.3390/ijerph7020427 20616983 PMC2872290

[30] Larsen FB, Ankersen PV, Poulsen S. Hvordan har du det? 2010. Sundhedsprofil for region og kommuner. Voksne. 2011. Aarhus: Center for Folkesundhed; 2011.

