# Supplementary material for: Non-melanoma skin cancer and risk of Alzheimer’s disease and all-cause dementia
Source: PLoS One. 2017 Feb 22;12(2):e0171527. doi: 10.1371/journal.pone.0171527 (PMC5321271; doi:10.1371/journal.pone.0171527)
Supplement: S1 Strobe — (DOC) [file pone.0171527.s001.doc]

**S1 STROBE Statement—Checklist of items that should be included in reports of cohort studies**

|  | Item No | Recommendation | Comment |
| --- | --- | --- | --- |
| **Title and abstract** | 1 | (*a*) Indicate the study’s design with a commonly used term in the title or the abstract | P2: Stated in abstract (“We conducted a nationwide cohort study”) |
|  |  | (*b*) Provide in the abstract an informative and balanced summary of what was done and what was found | P2–3: In abstract the background and main methods and findings are reported and interpreted taking limitations into account (e.g. “the observed association may represent confounding…”) |
| Introduction | | |  |
| Background/rationale | 2 | Explain the scientific background and rationale for the investigation being reported | P4: The scientific background, including discussion of previous studies in first and third paragraph and some biological explanations in the second paragraph (“A number of plausible biological factors link cancer to a decline in incident dementia. For example, decreased activity of tumor suppressor proteins and overexpression of Pin1 (peptidyl-prolyl cis-trans isomerase, NIMA-interacting 1) are associated both with cancer and with a lower risk of Alzheimer’s disease”). |
| Objectives | 3 | State specific objectives, including any prespecified hypotheses | P4: Stated in last paragraph of introduction (“To provide more accurate estimates of dementia risk within a general population cohort with long-term follow-up, we conducted a large matched cohort study of the association between NMSC and risk of Alzheimer’s disease (primary outcome) and all-cause dementia.”) |
| Methods | | |  |
| Study design | 4 | Present key elements of study design early in the paper | P4: Last paragraph of introduction states the design (“large matched cohort study”) |
| Setting | 5 | Describe the setting, locations, and relevant dates, including periods of recruitment, exposure, follow-up, and data collection | P5–7: Setting and data sources, including periods of data collection, are described in the subheading “Setting and data sources”. The study population, including sampling of the exposed cohort and the comparison cohort, is described in the Study population section (e.g. “From a source population of 6.9 million persons, we identified all adults aged 18 years or older with a first-time NMSC recorded in the DCR between 1 January 1980 and 30 November 2013.”) |
| Participants | 6 | (*a*) Give the eligibility criteria, and the sources and methods of selection of participants. Describe methods of follow-up | P6–7: The “study population” and “dementia” sections describe the eligibility criteria, data sources, and follow-up for dementia (e.g. “We used the DNPR and the PCRR to identify all inpatient and hospital outpatient clinic diagnoses of dementia among study participants following the index date.”). The first sentence in the Statistical analysis section also describes the periods of follow-up (“We followed participants from the index date until a diagnosis of dementia, death, emigration, or November 30, 2013, whichever came first.”) |
|  |  | (*b*) For matched studies, give matching criteria and number of exposed and unexposed | P6: Described as “We used the Civil Registration System to randomly select a comparison cohort of up to 5 individuals matched (with replacement) to each NMSC patient by sex and birth year.” |
| Variables | 7 | Clearly define all outcomes, exposures, predictors, potential confounders, and effect modifiers. Give diagnostic criteria, if applicable | See items 6 and 7 for exposure and outcome defintions. Potential confounders are described on P9 in the Comorbidity section beginning with “We identified participants history of hospital inpatient and outpatient diagnoses in the DNPR before or on the index date”. |
| Data sources/ measurement | 8* | For each variable of interest, give sources of data and details of methods of assessment (measurement). Describe comparability of assessment methods if there is more than one group | See items 5–7. |
| Bias | 9 | Describe any efforts to address potential sources of bias | P7–9: The Statistical analysis section describes adjustment for comorbidities (“adjusted for the individual cardiovascular diseases, risk factors for cardiovascular diseases, alcohol-related diagnoses, cancer, and multiple sclerosis”) and sensitivity analyses (“We examined the robustness of our results in several sensitivity analyses […]”) |
| Study size | 10 | Explain how the study size was arrived at | P6: Description of eligibility criteria for the cohort is described in the Study population section. |
| Quantitative variables | 11 | Explain how quantitative variables were handled in the analyses. If applicable, describe which groupings were chosen and why | NA. Age, which was a matching factor, was handled using stratified Cox (P9: “Cox proportional hazard regression stratified by matching factors”) |
| Statistical methods | 12 | (*a*) Describe all statistical methods, including those used to control for confounding | P7–9: The Statistical analysis section describes all methods used. |
|  |  | (*b*) Describe any methods used to examine subgroups and interactions | P8: Subgroup analyses are described in a paragraph of the Statistical analysis section beginning with “We performed several predefined subgroup analyses.” |
|  |  | (*c*) Explain how missing data were addressed | NA |
|  |  | (*d*) If applicable, explain how loss to follow-up was addressed | P7: In statistical analyses, follow-up is described: “We followed participants from the index date until a diagnosis of dementia, death, emigration, or November 30, 2013, whichever came first.” |
|  |  | (*e*) Describe any sensitivity analyses | See item 9. |
| Results | | |  |
| Participants | 13* | (a) Report numbers of individuals at each stage of study—eg numbers potentially eligible, examined for eligibility, confirmed eligible, included in the study, completing follow-up, and analysed | See item 10 about eligibility. |
|  |  | (b) Give reasons for non-participation at each stage | Non-participation is related only to the eligibility criteria. Besides age below 18 years and previous dementia diagnosis, no exclusions were performed. |
|  |  | (c) Consider use of a flow diagram | – |
| Descriptive data | 14* | (a) Give characteristics of study participants (eg demographic, clinical, social) and information on exposures and potential confounders | P9–10: Study cohorts are described in the first paragraph of the results and in table 1 and a supplementary table. |
|  |  | (b) Indicate number of participants with missing data for each variable of interest | NA |
|  |  | (c) Summarise follow-up time (eg, average and total amount) | Table 1 and a supplementary table for cohorts defined by NMSC subtypes. |
| Outcome data | 15* | Report numbers of outcome events or summary measures over time | Tables 2 and 3 |
| Main results | 16 | (*a*) Give unadjusted estimates and, if applicable, confounder-adjusted estimates and their precision (eg, 95% confidence interval). Make clear which confounders were adjusted for and why they were included | Tables 2 and 3 |
|  |  | (*b*) Report category boundaries when continuous variables were categorized | Table 1 |
|  |  | (*c*) If relevant, consider translating estimates of relative risk into absolute risk for a meaningful time period | Figures 1 and 2 an Results section (P12): “There was no overall difference in the risk of Alzheimer’s disease in the NMSC (4·6%, 95% CI: 4·4%–4·8%) and comparison cohorts (4·7%, 95% CI: 4·6%–4·9%) at the end of follow-up (34 years).” |
| Other analyses | 17 | Report other analyses done—eg analyses of subgroups and interactions, and sensitivity analyses | P16: Last paragraph of Results section describes results of subgroup and sensitivity analyses (e.g. “We observed no major differences in associations between NMSC and Alzheimer’s disease (table 3) or all-cause dementia (appendix p4) in subgroups defined by age, sex, calendar period of NMSC diagnosis, and comorbidities.”) |
| Discussion | | |  |
| Key results | 18 | Summarise key results with reference to study objectives | P19: First paragraph of discussion summarizes results (“Using registry data collected over a period of more than 40 years, we found that NMSC was associated with small reductions in relative risks of Alzheimer’s disease (5%) and all-cause dementia (8%), compared to individuals without NMSC. The absolute risk difference of Alzheimer’s disease was below 1% after ten years of follow-up.”) |
| Limitations | 19 | Discuss limitations of the study, taking into account sources of potential bias or imprecision. Discuss both direction and magnitude of any potential bias | P19–21: The majority of the discussion section includes a discussion of limitations, including potential survivor bias, selection bias, validity and completeness of dementia diagnoses, and confounding. |
| Interpretation | 20 | Give a cautious overall interpretation of results considering objectives, limitations, multiplicity of analyses, results from similar studies, and other relevant evidence | P22: Last paragraph of discussion includes a summary taking into account potential limitations (“However, the observed small effect sizes and the possibility of residual confounding from lifestyle factors associated with both Alzheimer’s risk and NMSC suggest the alternative interpretation that NMSC is unrelated to either Alzheimer’s disease or all-cause dementia.”) |
| Generalisability | 21 | Discuss the generalisability (external validity) of the study results | P21: Discussed when considering potential incompleteness of dementia (“may affect the generalizability of our results, but only if the association between NMSC and dementia or Alzheimer’s disease depends on dementia severity”) |
| Other information | | |  |
| Funding | 22 | Give the source of funding and the role of the funders for the present study and, if applicable, for the original study on which the present article is based | P22: “The study was supported by the Program for Clinical Research Infrastructure (PROCRIN) established by the Lundbeck Foundation and the Novo Nordisk Foundation, the Danish Cancer Society, and the Aarhus University Research Foundation.” |

*Give information separately for exposed and unexposed groups.

**Note:** An Explanation and Elaboration article discusses each checklist item and gives methodological background and published examples of transparent reporting. The STROBE checklist is best used in conjunction with this article (freely available on the Web sites of PLoS Medicine at http://www.plosmedicine.org/, Annals of Internal Medicine at http://www.annals.org/, and Epidemiology at http://www.epidem.com/). Information on the STROBE Initiative is available at http://www.strobe-statement.org.
