# Supplementary material for: Non-melanoma skin cancer and risk of Alzheimer’s disease and all-cause dementia
Source: PLoS One. 2017 Feb 22;12(2):e0171527. doi: 10.1371/journal.pone.0171527 (PMC5321271; doi:10.1371/journal.pone.0171527)
Supplement: S1 Table — (DOCX) [file pone.0171527.s002.docx]

**S1 Table. Registry codes used in the study**

| Non-melanoma skin cancer | ICD-8: 173.09-173.49 and 173.60-173.61; ICD-10: C44 |
| --- | --- |
| Squamous cell carcinoma | ICD-10: C44 together with ICD-O-3: 80513, 80703, 80713, 80723, 80733, 80743, 80753, 80763, 80783, 80833, 80843, 80943, 80953, or 85603 |
| Basal cell carcinoma | ICD-10: C44 together with ICD-O-3: 80903, 80913, 80923, 80933, 80973, 80983, or 81233 |
| Other | Remaining C44 codes |
| Dementia | All codes listed below |
| Alzheimer disease | ICD-8: 290.09, 290.10 and ICD-10: F00, G30 (includes G30, G30.0, G30.1, G30.8, G30.9) |
| Vascular dementia | ICD-8: 293.09, 293.19; ICD-10: F01 (includes F01.0x, F01.1x, F01.2x, F01.3x, F01.8X, F01.9x) |
| Other dementia | ICD-8: 094.19, 292.09, 290.11, 290.18, 290.19; ICD-10: F02, F03, F1x.73 (F10.73 through F19.73); G23.1; G31.0, G31.0A, G31.0B, G31.1, G31.8B, G31.8E, G31.85 |
| Alzheimer disease in sensitivity analysis | ICD-8: 290.09, 290.10 and ICD-10: F00, F03, G30 (includes G30, G30.0, G30.1, G30.8, G30.9) |
| Mild cognitive impairment and amnestic syndromes (not used to identify incidence of dementia) | ICD-8: 291.19; ICD-10: F04, F04.9, F05.1, F06.7 and F06.7x, F1x.6 (F10.6, F18.6, F19.6) |
| Non-melanoma skin cancer risk factors (exclusion criteria in sensitivity analysis) |  |
| Solid organ transplantation | Danish Classification of Surgical Procedures (1977-1995): 322.09, 322.29, 322.50, 356.09, 472.70, 472.79, 488.40, 488.49, 574.80, 574.90; Nordic Medico-Statistical Committee (NOMESCO) Classification of Surgical Procedures (1996-): FQ, GDG, JLE, JJC, KAS |
| Human immunodeficiency virus infection | ICD-8: 079.83, Y40.49, Y41.49; ICD-10: B20-B24. |
| Other cancer | ICD-8: 140-207, 275.59 (except 173.09-173.49); ICD-10: C00–C96 (except C44) |
| Xeroderma pigmentosum | ICD-10: Q82.1 |
| Nevoid basal cell carcinoma syndrome (Gorlin syndrome) | ICD-10: Q82.8W |
| Oculocutaneous albinism | ICD-10: E70.3B |
| Cardiovascular diseases and risk factors |  |
| Hospital-diagnosed obesity | ICD-8: 277; ICD-10: E65-E68 |
| Hypertension | ICD-8: 400-404; ICD-10: I10-I15 |
| Ischemic heart disease |  |
| Angina pectoris | ICD-8: 413; ICD-10: I20 |
| Myocardial infarction | ICD-8: 410; ICD-10: I21-I23 |
| Percutaneous coronary intervention | Treatment codes: KFNG, KFNF |
| Congestive heart failure | ICD-8: 427.09, 427.10; 427.11, 427.19, 428.99 782.49; ICD-10: I11.0, I13.0, I13.2, I50 |
| Peripheral arterial disease | ICD-8: 440; 441; 442; 443; 444; 445; ICD-10: I70; I71; I72; I73; I74; I77) |
| Diabetes | ICD-8: 249, 250; ICD-10: E10-E14 |
| Chronic pulmonary disease | ICD-8: 491-492; ICD-10: J41−J44 |
| Alcohol-related diagnoses | ICD-8: 291, 303, 571.09, 571.10, 577.10, 979.59; ICD-10: F10 (except F1073), G31.2, G62.1, G72.1, I42.6, K29.2, K70, K86.0, T50.0A, Z72.1 |
| Multiple sclerosis | ICD-8: 340; ICD-10: G35 |

Abbreviations: ICD = *International Classification of Disease*
