# Supplementary material for: Non-melanoma skin cancer and risk of Alzheimer’s disease and all-cause dementia
Source: PLoS One. 2017 Feb 22;12(2):e0171527. doi: 10.1371/journal.pone.0171527 (PMC5321271; doi:10.1371/journal.pone.0171527)
Supplement: S2 Table — (DOCX) [file pone.0171527.s003.docx]

**S2 Table. Selected characteristics of persons diagnosed with non-melanoma skin cancer and members of a matched comparison cohort, Denmark, 1980–2013**

|  | Basal cell carcinoma  (n=182,260) | Comparison cohort (n=911,300) | | | | Squamous cell carcinoma  (n=25,783) | | | | Comparison cohort (n=128,907) | |
| --- | --- | --- | --- | --- | --- | --- | --- | --- | --- | --- | --- |
| Age groups, years |  |  | | | |  | | | |  | |
| 18–49 | 24,978 (13.7) | 124,982 (13.7) | | | | 950 (3.7) | | | | 4,778 (3.7) | |
| 50–59 | 31,427 (17.2) | 157,340 (17.3) | | | | 2,042 (7.9) | | | | 10,189 (7.9) | |
| 60–74 | 73,116 (40.1) | 365,449 (40.1) | | | | 8,876 (34.4) | | | | 44,397 (34.4) | |
| 75–84 | 39,116 (21.5) | 195,239 (21.4) | | | | 8,669 (33.6) | | | | 43,440 (33.7) | |
| 85+ | 13,623 (7.5) | 68,290 (7.5) | | | | 5,246 (20.3) | | | | 26,103 (20.2) | |
| Sex |  |  | | | |  | | | |  | |
| Women | 95,397 (52.3) | 476,985 (52.3) | | | | 10,510 (40.8) | | | | 52,548 (40.8) | |
| Men | 86,863 (47.7) | 434,315 (47.7) | | | | 15,273 (59.2) | | | | 76,359 (59.2) | |
| Calendar period of skin cancer diagnosis | | | |  |  | | |  |  | | |
| 1980–1994 | 45,358 (24.9) | 226,790 (24.9) | | | | 7,311 (28.4) | | | | 36,551 (28.4) | |
| 1995–2003 | 42,523 (23.3) | 212,615 (23.3) | | | | 6,004 (23.3) | | | | 30,018 (23.3) | |
| 2004–2013 | 94,379 (51.8) | 471,895 (51.8) | | | | 12,468 (48.4) | | | | 62,338 (48.4) | |
| Comorbidities | |  |  | | | |  | | | |  |
| Hospital-diagnosed obesity | 3,070 (1.7) | 20,437 (2.2) | | | | 522 (2.0) | | | | 2,430 (1.9) | |
| Hypertension | 18,695 (10.3) | 89,176 (9.8) | | | | 3,907 (15.2) | | | | 16,065 (12.5) | |
| Ischemic heart disease | 14,009 (7.7) | 71,987 (7.9) | | | | 3,060 (11.9) | | | | 14,071 (10.9) | |
| Angina pectoris | 10,333 (5.7) | 51,377 (5.6) | | | | 2,171 (8.4) | | | | 9,614 (7.5) | |
| Myocardial infarction | 6,903 (3.8) | 37,987 (4.2) | | | | 1,676 (6.5) | | | | 7,971 (6.2) | |
| Percutaneous coronary intervention | 2,738 (1.5) | 14,009 (1.5) | | | | 500 (1.9) | | | | 2,498 (1.9) | |
| Congestive heart failure | 5,188 (2.8) | 28,034 (3.1) | | | | 1,585 (6.1) | | | | 6,742 (5.2) | |
| Peripheral arterial disease | 5,555 (3.0) | 28,776 (3.2) | | | | 1,404 (5.4) | | | | 5,833 (4.5) | |
| Diabetes | 6,642 (3.6) | 40,330 (4.4) | | | | 1,578 (6.1) | | | | 7,021 (5.4) | |
| Chronic obstructive pulmonary disease | 6,591 (3.6) | 36,955 (4.1) | | | | 1,607 (6.2) | | | | 7,093 (5.5) | |
| Alcohol-related disease | 2,570 (1.4) | 17,291 (1.9) | | | | 430 (1.7) | | | | 2,138 (1.7) | |
| Other cancer | 18,718 (10.3) | 68,942 (7.6) | | | | 3,513 (13.6) | | | | 12,229 (9.5) | |
| Multiple sclerosis | 443 (0.2) | 2,060 (0.2) | | | | 33 (0.1) | | | | 210 (0.2) | |
| Solid organ transplantation | 278 (0.2) | 246 (0.0) | | | | 167 (0.6) | | | | 20 (0.0) | |
| Human immunodeficiency virus infection | 90 (0.0) | 298 (0.0) | | | | 24 (0.1) | | | | 28 (0.0) | |
| Follow-up (years) |  |  | | | |  | | | |  | |
| Total (range) | 1,464,878 (0–34) | 7,048,853 (0–34) | | | | 153,569 (0–34) | | | | 819,735 (0–34) | |
| Median (interquartile range) | 6.1 (2.9–11.5) | 5.8 (2.6–11.0) | | | | 4.3 (1.8–8.3) | | | | 4.7 (2.0–8.9) | |

Data are presented as numbers (%). Xeroderma pigmentosum, nevoid basal cell carcinoma syndrome, and albinism were omitted from the table because of low prevalence (≤ 3 persons).
