# Supplementary material for: Non-melanoma skin cancer and risk of Alzheimer’s disease and all-cause dementia
Source: PLoS One. 2017 Feb 22;12(2):e0171527. doi: 10.1371/journal.pone.0171527 (PMC5321271; doi:10.1371/journal.pone.0171527)
Supplement: S3 Table — (DOCX) [file pone.0171527.s004.docx]

**S3 Table. Rates and hazard ratios of vascular dementia and other non-Alzheimer dementia in patients with non-melanoma skin cancer compared with a matched comparison cohort, Denmark 1980–2013**

|  | **NMSC cohort** | | **Matched cohort** | | **Unadjusted HR (95% CI)**† | **Adjusted HR (95% CI)**‡ |
| --- | --- | --- | --- | --- | --- | --- |
|  | **No. of events** | **Rate (95% CI)*** | **No. of events** | **Rate (95% CI)*** |  |  |
| **Any NMSC** |  |  |  |  |  |  |
| Vascular dementia | 1,457 | 0.87 (0.83–0.92) | 7,924 | 0.97 (0.95–1.00) | 0.86 (0.81–0.92) | 0.87 (0.82–0.92) |
| Other dementia | 6,045 | 3.63 (3.54–3.72) | 30,768 | 3.78 (3.74–3.82) | 0.92 (0.89–0.95) | 0.92 (0.89–0.95) |
| **Basal cell carcinoma** | | | | | | |
| Vascular dementia | 1,219 | 0.83 (0.79–0.88) | 6,565 | 0.93 (0.91–0.95) | 0.85 (0.80–0.91) | 0.86 (0.80–0.92) |
| Other dementia | 5,020 | 3.43 (3.33–3.52) | 24,930 | 3.54 (3.49–3.58) | 0.91 (0.88–0.94) | 0.91 (0.88–0.95) |
| **Squamous cell carcinoma** | | | | | | |
| Vascular dementia | 198 | 1.29 (1.11–1.47) | 1,114 | 1.36 (1.28–1.44) | 0.92 (0.78–1.09) | 0.93 (0.79–1.10) |
| Other dementia | 872 | 5.68 (5.30–6.06) | 4,954 | 6.04 (5.88–6.21) | 0.93 (0.86–1.01) | 0.92 (0.85–1.00) |
| Abbreviations: CI = confidence interval; HR = hazard ratio; NMSC = non-melanoma skin cancer  *Rate per 1,000 person-years.  †Hazard ratios calculated with stratified Cox proportional hazard regression adjusted by study design for age, sex, and calendar period of index date.  ‡Adjusted additionally for alcohol-related diagnoses, hospital-diagnosed obesity, hypertension, ischemic heart disease (angina pectoris, myocardial infarction, and percutaneous coronary intervention), congestive heart failure, peripheral artery disease, chronic pulmonary disease, diabetes, other cancer, and multiple sclerosis. | | | | | | |
