# Supplementary material for: Non-melanoma skin cancer and risk of Alzheimer’s disease and all-cause dementia
Source: PLoS One. 2017 Feb 22;12(2):e0171527. doi: 10.1371/journal.pone.0171527 (PMC5321271; doi:10.1371/journal.pone.0171527)
Supplement: S4 Table — (DOCX) [file pone.0171527.s005.docx]

**S4 Table. Rates and hazard ratios of all-cause dementia associated with a previous diagnosis of non-melanoma skin cancer, by study characteristics, Denmark 1980–2013**

|  | **Rate (95% CI)*** | | | **Unadjusted HR (95% CI)**† | **Adjusted HR (95% CI)**‡ |
| --- | --- | --- | --- | --- | --- |
|  | **NMSC cohort** | | **Comparison cohort** |  |  |
| **Age (years)** |  | |  |  |  |
| 18–49 | 0.36 (0.29–0.43) | | 0.46 (0.42–0.49) | 0.78 (0.63–0.96) | 0.79 (0.64–0.97) |
| 50–59 | 1.80 (1.66–1.94) | | 1.94 (1.88–2.01) | 0.89 (0.81–0.97) | 0.88 (0.81–0.97) |
| 60–74 | 6.93 (6.73–7.13) | | 7.02 (6.93–7.11) | 0.96 (0.92–0.99) | 0.96 (0.93–1.00) |
| 75–84 | 17.37 (16.88–17.87) | | 18.54 (18.30–18.77) | 0.92 (0.89–0.95) | 0.92 (0.89–0.95) |
| 85+ | 23.49 (22.33–24.64) | | 26.61 (26.05–27.17) | 0.86 (0.81–0.92) | 0.86 (0.81–0.92) |
| **Sex** |  | |  |  |  |
| Women | 7.27 (7.09–7.45) | | 7.71 (7.63–7.79) | 0.90 (0.88–0.93) | 0.91 (0.88–0.93) |
| Men | 6.73 (6.54–6.91) | | 6.92 (6.84–7.00) | 0.94 (0.91–0.98) | 0.94 (0.91–0.97) |
| **Calendar period of NMSC diagnosis** |  | |  |  |  |
| 1980–1994 | 6.78 (6.59–6.97) | | 6.82 (6.73–6.90) | 0.97 (0.93–1.00) | 0.97 (0.93–1.00) |
| 1995–2003 | 7.78 (7.54–8.03) | | 8.16 (8.04–8.27) | 0.91 (0.88–0.95) | 0.91 (0.88–0.95) |
| 2004–2013 | 6.53 (6.30–6.77) | | 7.25 (7.14–7.37) | 0.88 (0.84–0.91) | 0.88 (0.85–0.92) |
| **Any history of cardiovascular diseases or risk factors** | |  |  |  |  |
| Yes | 11.05 (10.63–11.48) | | 11.76 (11.56–11.96) | 0.89 (0.85–0.93) | 0.89 (0.86–0.93) |
| No | 6.34 (6.21–6.47) | | 6.59 (6.53–6.66) | 0.93 (0.91–0.96) | 0.93 (0.91–0.96) |
| **Any history of alcohol-related disease** |  | |  |  |  |
| Yes | 8.89 (7.44–10.34) | | 10.08 (9.46–10.70) | 0.82 (0.69–0.97) | 0.81 (0.68–0.97) |
| No | 6.99 (6.87–7.12) | | 7.30 (7.24–7.36) | 0.93 (0.91–0.95) | 0.93 (0.91–0.94) |
| **Any history of other cancer** |  | |  |  |  |
| Yes | 9.05 (8.53–9.58) | | 10.84 (10.52–11.17) | 0.91 (0.85–0.97) | 0.91 (0.85–0.97) |
| No | 6.85 (6.72–6.98) | | 7.16 (7.10–7.22) | 0.93 (0.91–0.94) | 0.92 (0.91–0.94) |

Abbreviations: CI = confidence interval; HR = hazard ratio; NMSC = non-melanoma skin cancer

*Rate per 1,000 person-years.

†Computed using stratified Cox proportional hazard regression adjusted by study design for age, sex, and calendar period of the skin cancer diagnosis/index date. In analyses stratified by comorbidities, conventional Cox regression was used with additional adjustment for matching factors.

‡Adjusted additionally for alcohol-related diagnoses, hospital-diagnosed obesity, hypertension, ischemic heart disease (angina pectoris, myocardial infarction, and percutaneous coronary intervention), congestive heart failure, peripheral artery disease, chronic pulmonary disease, diabetes, other cancer, and multiple sclerosis.
