# Supplementary material for: Non-melanoma skin cancer and risk of Alzheimer’s disease and all-cause dementia
Source: PLoS One. 2017 Feb 22;12(2):e0171527. doi: 10.1371/journal.pone.0171527 (PMC5321271; doi:10.1371/journal.pone.0171527)
Supplement: S5 Table — Sensitivity analysis excluding persons with previous cancer diagnosis, solid organ transplantation, HIV infection, xeroderma pigmentosum, nevoid basal cell carcinoma syndrome, and/or albinism. (DOCX) [file pone.0171527.s006.docx]

**S5 Table. Adjusted hazard ratios (95% confidence intervals)* of dementia associated with a previous diagnosis of non-melanoma skin cancer, Denmark 1980–2013. Sensitivity analysis excluding persons with previous cancer diagnosis, solid organ transplantation, HIV infection, xeroderma pigmentosum, nevoid basal cell carcinoma syndrome, and/or albinism**

| **All-cause dementia** |  |
| --- | --- |
| NMSC overall | 0.93 (0.90–0.95) |
| Basal cell carcinoma | 0.92 (0.90–0.94) |
| Squamous cell carcinoma | 0.95 (0.89–1.01) |
| **Alzheimer disease** |  |
| NMSC overall | 0.95 (0.92–0.99) |
| Basal cell carcinoma | 0.95 (0.91–0.99) |
| Squamous cell carcinoma | 0.98 (0.88–1.09) |
| **Vascular dementia** |  |
| NMSC overall | 0.88 (0.82–0.94) |
| Basal cell carcinoma | 0.87 (0.81–0.93) |
| Squamous cell carcinoma | 0.94 (0.78–1.12) |
| **Other dementia** |  |
| NMSC overall | 0.92 (0.89–0.95) |
| Basal cell carcinoma | 0.91 (0.88–0.95) |
| Squamous cell carcinoma | 0.93 (0.85–1.01) |

*Adjusting for alcohol-related diagnoses, hospital-diagnosed obesity, hypertension, ischemic heart disease (angina pectoris, myocardial infarction, and percutaneous coronary intervention), congestive heart failure, peripheral artery disease, chronic pulmonary disease, diabetes, and multiple sclerosis. Computed using stratified Cox proportional hazard regression adjusted by study design for age, sex, and calendar period of the skin cancer diagnosis/index date.
