# Supplementary material for: Non-melanoma skin cancer and risk of Alzheimer’s disease and all-cause dementia
Source: PLoS One. 2017 Feb 22;12(2):e0171527. doi: 10.1371/journal.pone.0171527 (PMC5321271; doi:10.1371/journal.pone.0171527)
Supplement: S6 Table — Sensitivity analysis adjusting for alcohol-related disease and cardiovascular diseases and risk factors as time-varying covariates. (DOCX) [file pone.0171527.s007.docx]

**S6 Table. Adjusted hazard ratios (95% confidence interval)* of dementia associated with a previous diagnosis of non-melanoma skin cancer, Denmark 1980–2013. Sensitivity analysis adjusting for alcohol-related disease and cardiovascular diseases and risk factors as time-varying covariates**

| **All-cause dementia** |  |
| --- | --- |
| NMSC overall | 0.92 (0.90–0.94) |
| Basal cell carcinoma | 0.91 (0.89–0.94) |
| Squamous cell carcinoma | 0.94 (0.88–0.99) |
| **Alzheimer disease** |  |
| NMSC overall | 0.95 (0.91–0.98) |
| Basal cell carcinoma | 0.95 (0.91–0.98) |
| Squamous cell carcinoma | 0.95 (0.86–1.05) |
| **Vascular dementia** |  |
| NMSC overall | 0.86 (0.81–0.92) |
| Basal cell carcinoma | 0.85 (0.80–0.91) |
| Squamous cell carcinoma | 0.93 (0.78–1.10) |
| **Other dementia** |  |
| NMSC overall | 0.92 (0.89–0.95) |
| Basal cell carcinoma | 0.91 (0.88–0.94) |
| Squamous cell carcinoma | 0.93 (0.85–1.01) |

*Adjusting for other cancer, multiple sclerosis, and as time varying covariates: alcohol-related diagnoses, hospital-diagnosed obesity, hypertension, ischemic heart disease (angina pectoris, myocardial infarction, and percutaneous coronary intervention), congestive heart failure, peripheral artery disease, chronic pulmonary disease, diabetes. Computed using stratified Cox proportional hazard regression adjusted by study design for age, sex, and calendar period of the skin cancer diagnosis/index date
