# Supplementary material for: Non-melanoma skin cancer and risk of Alzheimer’s disease and all-cause dementia
Source: PLoS One. 2017 Feb 22;12(2):e0171527. doi: 10.1371/journal.pone.0171527 (PMC5321271; doi:10.1371/journal.pone.0171527)
Supplement: S7 Table — Sensitivity analysis including unspecified dementia diagnoses in the classification of Alzheimer disease*. (DOCX) [file pone.0171527.s008.docx]

**S7 Table. Rates and hazard ratios of Alzheimer disease in patients with non-melanoma skin cancer compared with members of a matched comparison cohort, Denmark, 1980–2013. Sensitivity analysis including unspecified dementia diagnoses in the classification of Alzheimer disease***

|  | **NMSC cohort** | | **Comparison cohort** | | **Unadjusted HR (95% CI)**‡ | **Adjusted HR (95% CI)**§ |
| --- | --- | --- | --- | --- | --- | --- |
|  | **No. of events** | **Rate (95% CI)**† | **No. of events** | **Rate (95% CI)**† |  |  |
| Any NMSC | 9,632 | 5.78 (5.67–5.90) | 49,121 | 6.04 (5.99–6.09) | 0.92 (0.90–0.94) | 0.92 (0.90–0.95) |
| Basal cell carcinoma | 8,031 | 5.48 (5.36–5.60) | 39,844 | 5.65 (5.60–5.71) | 0.92 (0.89–0.94) | 0.92 (0.90–0.95) |
| Squamous cell carcinoma | 1,353 | 8.81 (8.34–9.28) | 7,781 | 9.49 (9.28–9.70) | 0.92 (0.87–0.99) | 0.92 (0.86–0.98) |

Abbreviations: CI = confidence interval; HR = hazard ratio; NMSC = non-melanoma skin cancer

*****International Classification of Diseases codes are given in table e-1.

†Rate per 1,000 person-years.

‡Computed using stratified Cox proportional hazard regression adjusted by study design for age, sex, and calendar period of the skin cancer diagnosis/index date.

§Adjusted additionally for alcohol-related diagnoses, hospital-diagnosed obesity, hypertension, ischemic heart disease (angina pectoris, myocardial infarction, and percutaneous coronary intervention), congestive heart failure, peripheral artery disease, chronic pulmonary disease, diabetes, other cancer, and multiple sclerosis.
